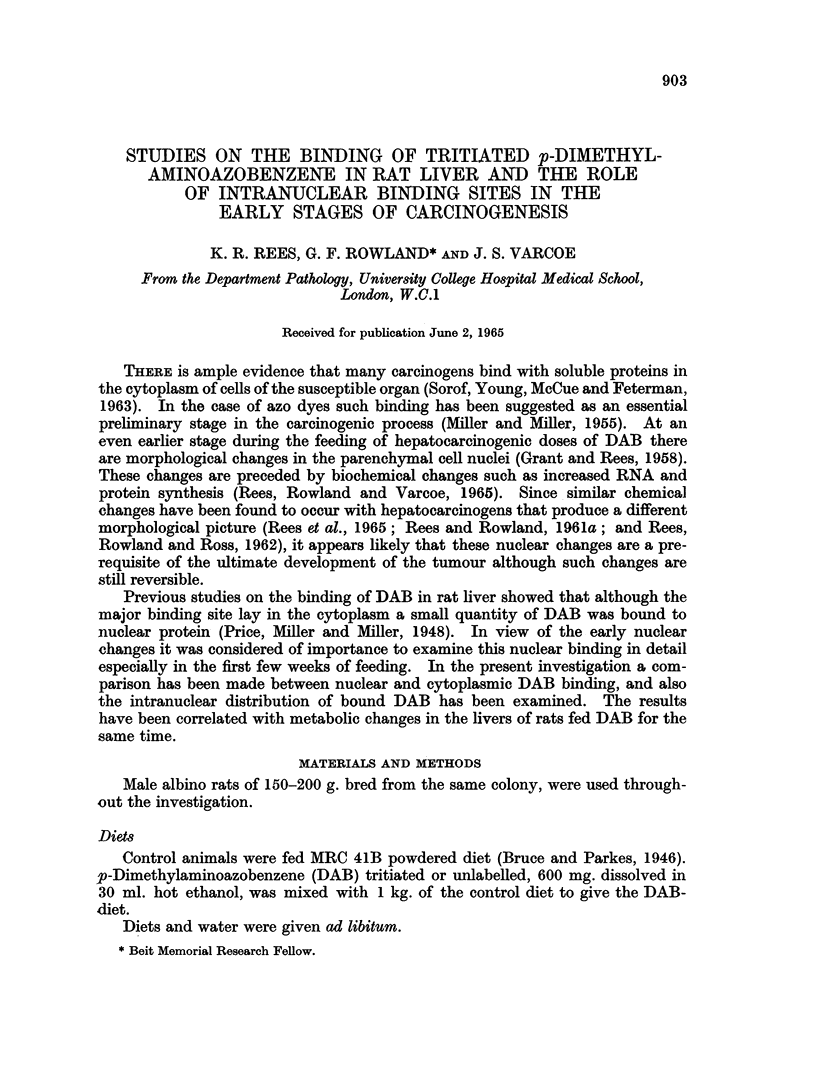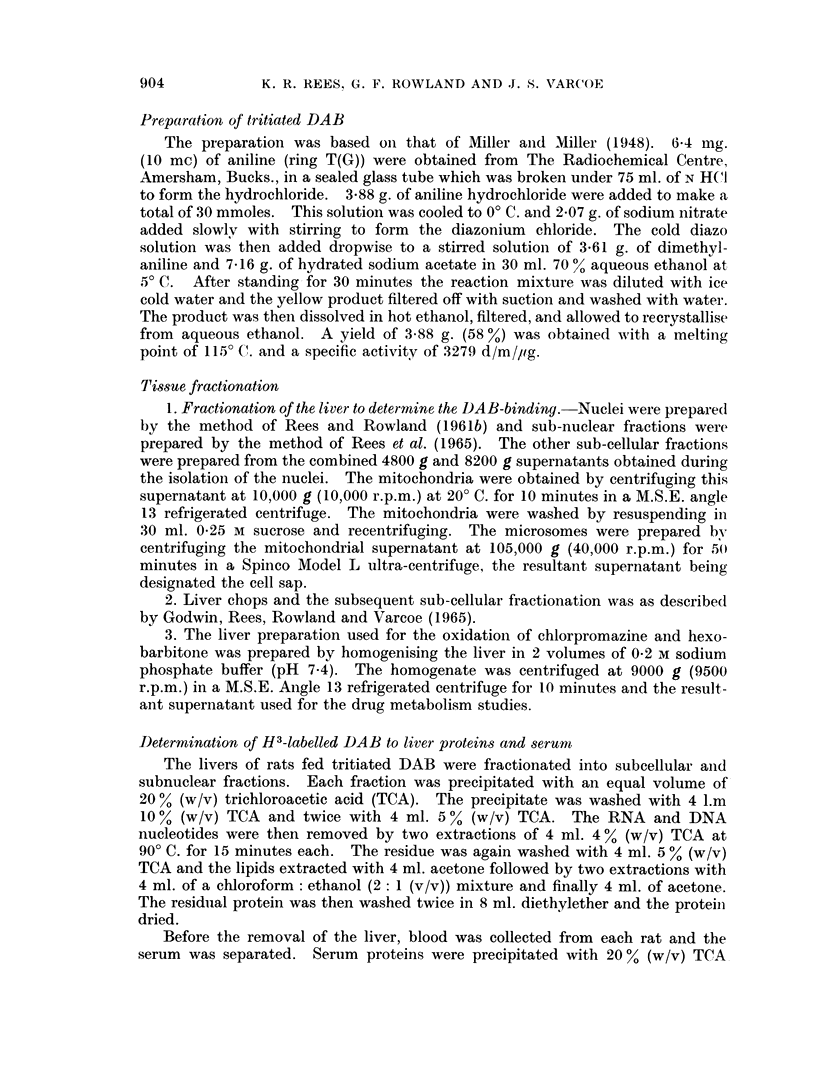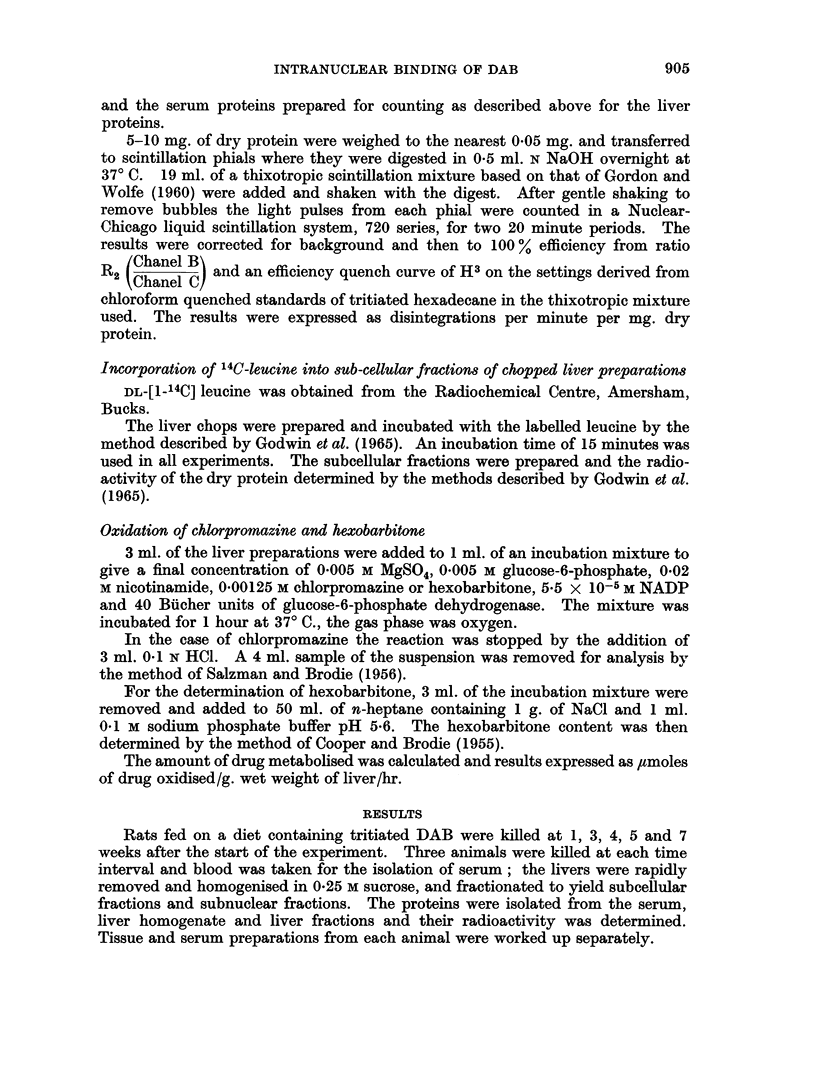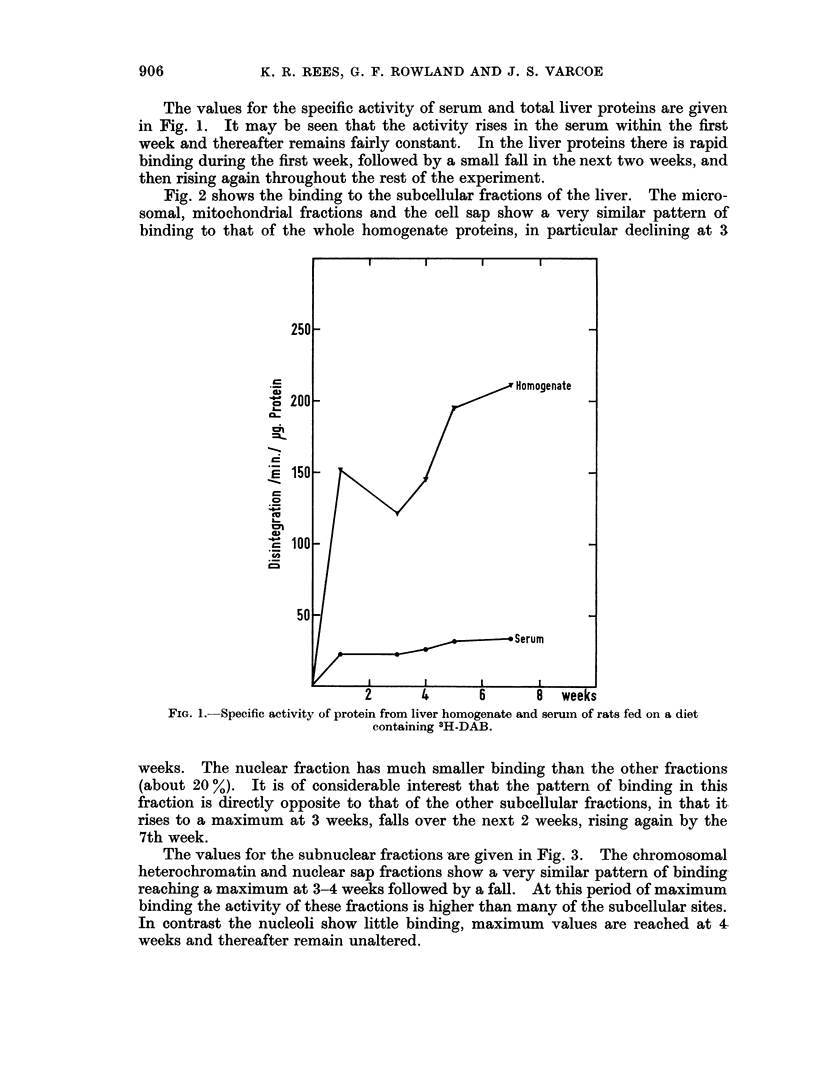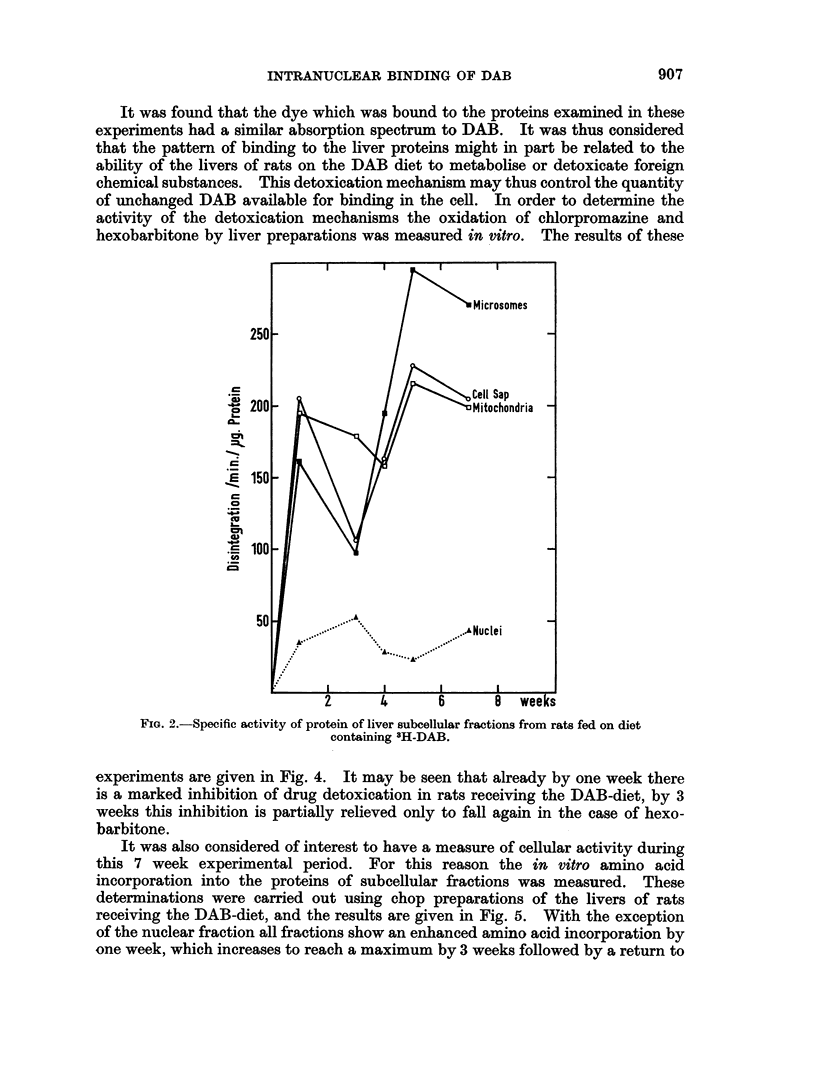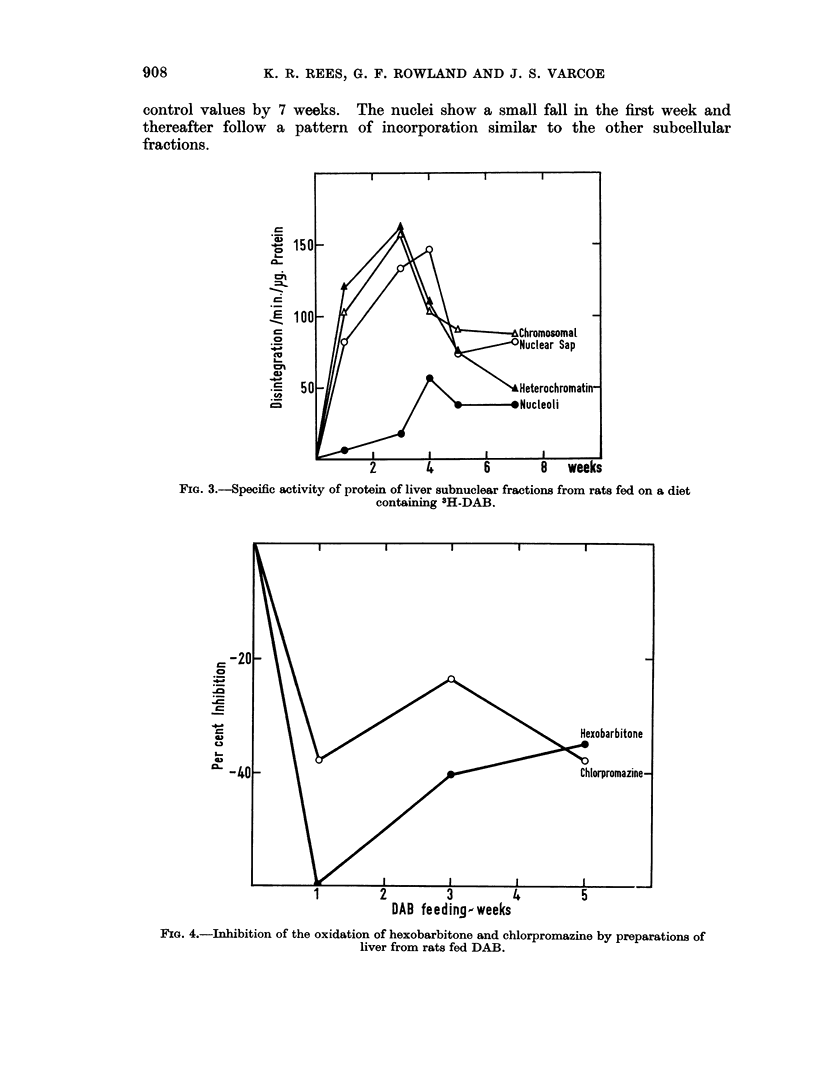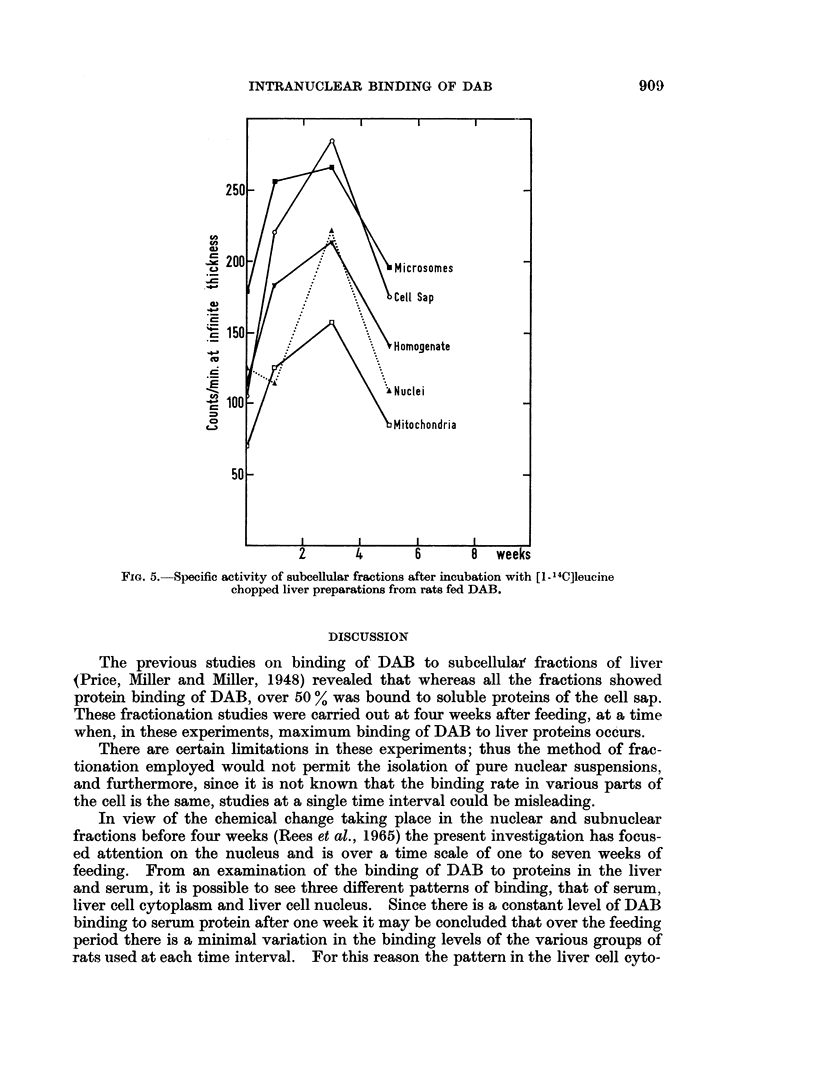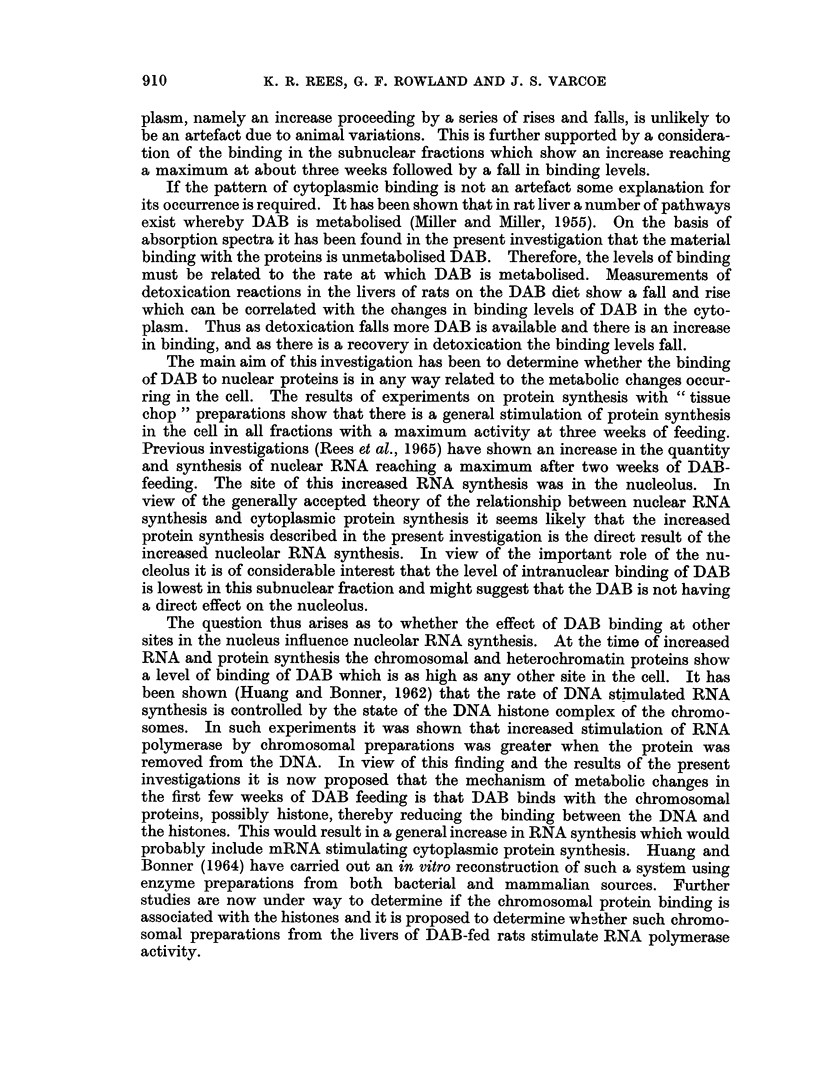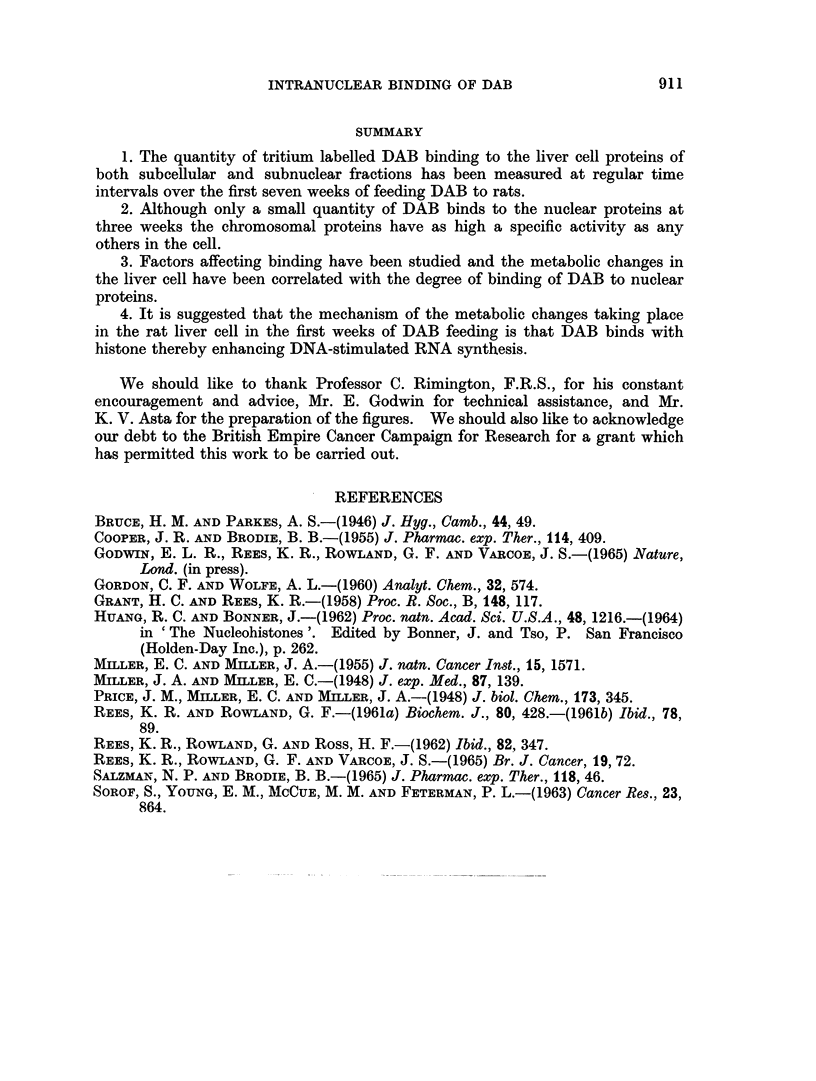# Studies on the binding of tritiated rho-dimethylaminoazobenzene in rat liver and the role of intranuclear binding sites in the early stages of carcinogenesis.

**DOI:** 10.1038/bjc.1965.105

**Published:** 1965-12

**Authors:** K. R. Rees, G. F. Rowland, J. S. Varcoe


### STUDIES ON THE BINDING OF TRITIATED *p*-DIMETHYLAMINOAZOBENZENE IN RAT LIVER AND THE ROLE OF INTRANUCLEAR BINDING SITES IN THE EARLY STAGES OF CARCINOGENESIS

K. R. REES, G. F. ROWLAND* AND J. S. VARCOE

*From the Department Pathology, University College Hospital Medical School, London, W.C.1*

Received for publication June 2, 1965

THERE is ample evidence that many carcinogens bind with soluble proteins in the cytoplasm of cells of the susceptible organ (Sorof, Young, McCue and Feterman, 1963). In the case of azo dyes such binding has been suggested as an essential preliminary stage in the carcinogenic process (Miller and Miller, 1955). At an even earlier stage during the feeding of hepatocarcinogenic doses of DAB there are morphological changes in the parenchymal cell nuclei (Grant and Rees, 1958). These changes are preceded by biochemical changes such as increased RNA and protein synthesis (Rees, Rowland and Varcoe, 1965). Since similar chemical changes have been found to occur with hepatocarcinogens that produce a different morphological picture (Rees *et al.*, 1965 ; Rees and Rowland, 1961*a* ; and Rees, Rowland and Ross, 1962), it appears likely that these nuclear changes are a prerequisite of the ultimate development of the tumour although such changes are still reversible.

Previous studies on the binding of DAB in rat liver showed that although the major binding site lay in the cytoplasm a small quantity of DAB was bound to nuclear protein (Price, Miller and Miller, 1948). In view of the early nuclear changes it was considered of importance to examine this nuclear binding in detail especially in the first few weeks of feeding. In the present investigation a comparison has been made between nuclear and cytoplasmic DAB binding, and also the intranuclear distribution of bound DAB has been examined. The results have been correlated with metabolic changes in the livers of rats fed DAB for the same time.

### MATERIALS AND METHODS

Male albino rats of 150–200 g. bred from the same colony, were used throughout the investigation.

### *Diets*

Control animals were fed MRC 41B powdered diet (Bruce and Parkes, 1946). *p*-Dimethylaminoazobenzene (DAB) tritiated or unlabelled, 600 mg. dissolved in 30 ml. hot ethanol, was mixed with 1 kg. of the control diet to give the DAB-diet.

Diets and water were given *ad libitum.*

* Beit Memorial Research Fellow.

*Preparation of tritiated DAB*

The preparation was based on that of Miller and Miller (1948). 6·4 mg. (10 mc) of aniline (ring T(G)) were obtained from The Radiochemical Centre, Amersham, Bucks., in a sealed glass tube which was broken under 75 ml. of N HCl to form the hydrochloride. 3·88 g. of aniline hydrochloride were added to make a total of 30 mmoles. This solution was cooled to 0° C. and 2·07 g. of sodium nitrate added slowly with stirring to form the diazonium chloride. The cold diazo solution was then added dropwise to a stirred solution of 3·61 g. of dimethyl-aniline and 7·16 g. of hydrated sodium acetate in 30 ml. 70 % aqueous ethanol at 5° C. After standing for 30 minutes the reaction mixture was diluted with ice cold water and the yellow product filtered off with suction and washed with water. The product was then dissolved in hot ethanol, filtered, and allowed to recrystallise from aqueous ethanol. A yield of 3·88 g. (58 %) was obtained with a melting point of 115° C. and a specific activity of 3279 d/m/$\mu$g.

*Tissue fractionation*

1. *Fractionation of the liver to determine the DAB-binding.*—Nuclei were prepared by the method of Rees and Rowland (1961*b*) and sub-nuclear fractions were prepared by the method of Rees *et al.* (1965). The other sub-cellular fractions were prepared from the combined 4800 *g* and 8200 *g* supernatants obtained during the isolation of the nuclei. The mitochondria were obtained by centrifuging this supernatant at 10,000 *g* (10,000 r.p.m.) at 20° C. for 10 minutes in a M.S.E. angle 13 refrigerated centrifuge. The mitochondria were washed by resuspending in 30 ml. 0·25 M sucrose and recentrifuging. The microsomes were prepared by centrifuging the mitochondrial supernatant at 105,000 *g* (40,000 r.p.m.) for 50 minutes in a Spinco Model L ultra-centrifuge, the resultant supernatant being designated the cell sap.

2. Liver chops and the subsequent sub-cellular fractionation was as described by Godwin, Rees, Rowland and Varcoe (1965).

3. The liver preparation used for the oxidation of chlorpromazine and hexobarbitone was prepared by homogenising the liver in 2 volumes of 0·2 M sodium phosphate buffer (pH 7·4). The homogenate was centrifuged at 9000 *g* (9500 r.p.m.) in a M.S.E. Angle 13 refrigerated centrifuge for 10 minutes and the resultant supernatant used for the drug metabolism studies.

*Determination of $H^3$-labelled DAB to liver proteins and serum*

The livers of rats fed tritiated DAB were fractionated into subcellular and subnuclear fractions. Each fraction was precipitated with an equal volume of 20 % (w/v) trichloroacetic acid (TCA). The precipitate was washed with 4 l.m 10 % (w/v) TCA and twice with 4 ml. 5 % (w/v) TCA. The RNA and DNA nucleotides were then removed by two extractions of 4 ml. 4 % (w/v) TCA at 90° C. for 15 minutes each. The residue was again washed with 4 ml. 5 % (w/v) TCA and the lipids extracted with 4 ml. acetone followed by two extractions with 4 ml. of a chloroform : ethanol (2 : 1 (v/v)) mixture and finally 4 ml. of acetone. The residual protein was then washed twice in 8 ml. diethylether and the protein dried.

Before the removal of the liver, blood was collected from each rat and the serum was separated. Serum proteins were precipitated with 20 % (w/v) TCA

and the serum proteins prepared for counting as described above for the liver proteins.

5–10 mg. of dry protein were weighed to the nearest 0·05 mg. and transferred to scintillation phials where they were digested in 0·5 ml. N NaOH overnight at 37° C. 19 ml. of a thixotropic scintillation mixture based on that of Gordon and Wolfe (1960) were added and shaken with the digest. After gentle shaking to remove bubbles the light pulses from each phial were counted in a Nuclear-Chicago liquid scintillation system, 720 series, for two 20 minute periods. The results were corrected for background and then to 100% efficiency from ratio $R_2 \left(\frac{\text{Chanel B}}{\text{Chanel C}}\right)$ and an efficiency quench curve of $H^3$ on the settings derived from chloroform quenched standards of tritiated hexadecane in the thixotropic mixture used. The results were expressed as disintegrations per minute per mg. dry protein.

*Incorporation of $^{14}$C-leucine into sub-cellular fractions of chopped liver preparations*

DL-[1-$^{14}$C] leucine was obtained from the Radiochemical Centre, Amersham, Bucks.

The liver chops were prepared and incubated with the labelled leucine by the method described by Godwin *et al.* (1965). An incubation time of 15 minutes was used in all experiments. The subcellular fractions were prepared and the radioactivity of the dry protein determined by the methods described by Godwin *et al.* (1965).

*Oxidation of chlorpromazine and hexobarbitone*

3 ml. of the liver preparations were added to 1 ml. of an incubation mixture to give a final concentration of 0·005 M $MgSO_4$, 0·005 M glucose-6-phosphate, 0·02 M nicotinamide, 0·00125 M chlorpromazine or hexobarbitone, $5{\cdot}5 \times 10^{-5}$ M NADP and 40 Bücher units of glucose-6-phosphate dehydrogenase. The mixture was incubated for 1 hour at 37° C., the gas phase was oxygen.

In the case of chlorpromazine the reaction was stopped by the addition of 3 ml. 0·1 N HCl. A 4 ml. sample of the suspension was removed for analysis by the method of Salzman and Brodie (1956).

For the determination of hexobarbitone, 3 ml. of the incubation mixture were removed and added to 50 ml. of *n*-heptane containing 1 g. of NaCl and 1 ml. 0·1 M sodium phosphate buffer pH 5·6. The hexobarbitone content was then determined by the method of Cooper and Brodie (1955).

The amount of drug metabolised was calculated and results expressed as $\mu$moles of drug oxidised/g. wet weight of liver/hr.

### RESULTS

Rats fed on a diet containing tritiated DAB were killed at 1, 3, 4, 5 and 7 weeks after the start of the experiment. Three animals were killed at each time interval and blood was taken for the isolation of serum ; the livers were rapidly removed and homogenised in 0·25 M sucrose, and fractionated to yield subcellular fractions and subnuclear fractions. The proteins were isolated from the serum, liver homogenate and liver fractions and their radioactivity was determined. Tissue and serum preparations from each animal were worked up separately.

The values for the specific activity of serum and total liver proteins are given in Fig. 1. It may be seen that the activity rises in the serum within the first week and thereafter remains fairly constant. In the liver proteins there is rapid binding during the first week, followed by a small fall in the next two weeks, and then rising again throughout the rest of the experiment.

Fig. 2 shows the binding to the subcellular fractions of the liver. The microsomal, mitochondrial fractions and the cell sap show a very similar pattern of binding to that of the whole homogenate proteins, in particular declining at 3

FIG. 1.—Specific activity of protein from liver homogenate and serum of rats fed on a diet containing $^{3}H$-DAB.

weeks. The nuclear fraction has much smaller binding than the other fractions (about 20 %). It is of considerable interest that the pattern of binding in this fraction is directly opposite to that of the other subcellular fractions, in that it rises to a maximum at 3 weeks, falls over the next 2 weeks, rising again by the 7th week.

The values for the subnuclear fractions are given in Fig. 3. The chromosomal heterochromatin and nuclear sap fractions show a very similar pattern of binding reaching a maximum at 3–4 weeks followed by a fall. At this period of maximum binding the activity of these fractions is higher than many of the subcellular sites. In contrast the nucleoli show little binding, maximum values are reached at 4 weeks and thereafter remain unaltered.

It was found that the dye which was bound to the proteins examined in these experiments had a similar absorption spectrum to DAB. It was thus considered that the pattern of binding to the liver proteins might in part be related to the ability of the livers of rats on the DAB diet to metabolise or detoxicate foreign chemical substances. This detoxication mechanism may thus control the quantity of unchanged DAB available for binding in the cell. In order to determine the activity of the detoxication mechanisms the oxidation of chlorpromazine and hexobarbitone by liver preparations was measured *in vitro*. The results of these

FIG. 2.—Specific activity of protein of liver subcellular fractions from rats fed on diet containing $^{3}$H-DAB.

experiments are given in Fig. 4. It may be seen that already by one week there is a marked inhibition of drug detoxication in rats receiving the DAB-diet, by 3 weeks this inhibition is partially relieved only to fall again in the case of hexobarbitone.

It was also considered of interest to have a measure of cellular activity during this 7 week experimental period. For this reason the *in vitro* amino acid incorporation into the proteins of subcellular fractions was measured. These determinations were carried out using chop preparations of the livers of rats receiving the DAB-diet, and the results are given in Fig. 5. With the exception of the nuclear fraction all fractions show an enhanced amino acid incorporation by one week, which increases to reach a maximum by 3 weeks followed by a return to control values by 7 weeks. The nuclei show a small fall in the first week and thereafter follow a pattern of incorporation similar to the other subcellular fractions.

FIG. 3.—Specific activity of protein of liver subnuclear fractions from rats fed on a diet containing $^3$H-DAB.

FIG. 4.—Inhibition of the oxidation of hexobarbitone and chlorpromazine by preparations of liver from rats fed DAB.

FIG. 5.—Specific activity of subcellular fractions after incubation with [1-$^{14}$C]leucine chopped liver preparations from rats fed DAB.

### DISCUSSION

The previous studies on binding of DAB to subcellular fractions of liver (Price, Miller and Miller, 1948) revealed that whereas all the fractions showed protein binding of DAB, over 50% was bound to soluble proteins of the cell sap. These fractionation studies were carried out at four weeks after feeding, at a time when, in these experiments, maximum binding of DAB to liver proteins occurs.

There are certain limitations in these experiments; thus the method of fractionation employed would not permit the isolation of pure nuclear suspensions, and furthermore, since it is not known that the binding rate in various parts of the cell is the same, studies at a single time interval could be misleading.

In view of the chemical change taking place in the nuclear and subnuclear fractions before four weeks (Rees *et al.*, 1965) the present investigation has focused attention on the nucleus and is over a time scale of one to seven weeks of feeding. From an examination of the binding of DAB to proteins in the liver and serum, it is possible to see three different patterns of binding, that of serum, liver cell cytoplasm and liver cell nucleus. Since there is a constant level of DAB binding to serum protein after one week it may be concluded that over the feeding period there is a minimal variation in the binding levels of the various groups of rats used at each time interval. For this reason the pattern in the liver cell cytoplasm, namely an increase proceeding by a series of rises and falls, is unlikely to be an artefact due to animal variations. This is further supported by a consideration of the binding in the subnuclear fractions which show an increase reaching a maximum at about three weeks followed by a fall in binding levels.

If the pattern of cytoplasmic binding is not an artefact some explanation for its occurrence is required. It has been shown that in rat liver a number of pathways exist whereby DAB is metabolised (Miller and Miller, 1955). On the basis of absorption spectra it has been found in the present investigation that the material binding with the proteins is unmetabolised DAB. Therefore, the levels of binding must be related to the rate at which DAB is metabolised. Measurements of detoxication reactions in the livers of rats on the DAB diet show a fall and rise which can be correlated with the changes in binding levels of DAB in the cytoplasm. Thus as detoxication falls more DAB is available and there is an increase in binding, and as there is a recovery in detoxication the binding levels fall.

The main aim of this investigation has been to determine whether the binding of DAB to nuclear proteins is in any way related to the metabolic changes occurring in the cell. The results of experiments on protein synthesis with " tissue chop " preparations show that there is a general stimulation of protein synthesis in the cell in all fractions with a maximum activity at three weeks of feeding. Previous investigations (Rees *et al.*, 1965) have shown an increase in the quantity and synthesis of nuclear RNA reaching a maximum after two weeks of DAB-feeding. The site of this increased RNA synthesis was in the nucleolus. In view of the generally accepted theory of the relationship between nuclear RNA synthesis and cytoplasmic protein synthesis it seems likely that the increased protein synthesis described in the present investigation is the direct result of the increased nucleolar RNA synthesis. In view of the important role of the nucleolus it is of considerable interest that the level of intranuclear binding of DAB is lowest in this subnuclear fraction and might suggest that the DAB is not having a direct effect on the nucleolus.

The question thus arises as to whether the effect of DAB binding at other sites in the nucleus influence nucleolar RNA synthesis. At the time of increased RNA and protein synthesis the chromosomal and heterochromatin proteins show a level of binding of DAB which is as high as any other site in the cell. It has been shown (Huang and Bonner, 1962) that the rate of DNA stimulated RNA synthesis is controlled by the state of the DNA histone complex of the chromosomes. In such experiments it was shown that increased stimulation of RNA polymerase by chromosomal preparations was greater when the protein was removed from the DNA. In view of this finding and the results of the present investigations it is now proposed that the mechanism of metabolic changes in the first few weeks of DAB feeding is that DAB binds with the chromosomal proteins, possibly histone, thereby reducing the binding between the DNA and the histones. This would result in a general increase in RNA synthesis which would probably include mRNA stimulating cytoplasmic protein synthesis. Huang and Bonner (1964) have carried out an *in vitro* reconstruction of such a system using enzyme preparations from both bacterial and mammalian sources. Further studies are now under way to determine if the chromosomal protein binding is associated with the histones and it is proposed to determine whether such chromosomal preparations from the livers of DAB-fed rats stimulate RNA polymerase activity.

SUMMARY

1. The quantity of tritium labelled DAB binding to the liver cell proteins of both subcellular and subnuclear fractions has been measured at regular time intervals over the first seven weeks of feeding DAB to rats.

2. Although only a small quantity of DAB binds to the nuclear proteins at three weeks the chromosomal proteins have as high a specific activity as any others in the cell.

3. Factors affecting binding have been studied and the metabolic changes in the liver cell have been correlated with the degree of binding of DAB to nuclear proteins.

4. It is suggested that the mechanism of the metabolic changes taking place in the rat liver cell in the first weeks of DAB feeding is that DAB binds with histone thereby enhancing DNA-stimulated RNA synthesis.

We should like to thank Professor C. Rimington, F.R.S., for his constant encouragement and advice, Mr. E. Godwin for technical assistance, and Mr. K. V. Asta for the preparation of the figures. We should also like to acknowledge our debt to the British Empire Cancer Campaign for Research for a grant which has permitted this work to be carried out.

REFERENCES

Bruce, H. M. and Parkes, A. S.—(1946) *J. Hyg., Camb.*, **44**, 49.

Cooper, J. R. and Brodie, B. B.—(1955) *J. Pharmac. exp. Ther.*, **114**, 409.

Godwin, E. L. R., Rees, K. R., Rowland, G. F. and Varcoe, J. S.—(1965) *Nature, Lond.* (in press).

Gordon, C. F. and Wolfe, A. L.—(1960) *Analyt. Chem.*, **32**, 574.

Grant, H. C. and Rees, K. R.—(1958) *Proc. R. Soc.*, B, **148**, 117.

Huang, R. C. and Bonner, J.—(1962) *Proc. natn. Acad. Sci. U.S.A.*, **48**, 1216.—(1964) in 'The Nucleohistones'. Edited by Bonner, J. and Tso, P. San Francisco (Holden-Day Inc.), p. 262.

Miller, E. C. and Miller, J. A.—(1955) *J. natn. Cancer Inst.*, **15**, 1571.

Miller, J. A. and Miller, E. C.—(1948) *J. exp. Med.*, **87**, 139.

Price, J. M., Miller, E. C. and Miller, J. A.—(1948) *J. biol. Chem.*, **173**, 345.

Rees, K. R. and Rowland, G. F.—(1961*a*) *Biochem. J.*, **80**, 428.—(1961*b*) *Ibid.*, **78**, 89.

Rees, K. R., Rowland, G. and Ross, H. F.—(1962) *Ibid.*, **82**, 347.

Rees, K. R., Rowland, G. F. and Varcoe, J. S.—(1965) *Br. J. Cancer*, **19**, 72.

Salzman, N. P. and Brodie, B. B.—(1965) *J. Pharmac. exp. Ther.*, **118**, 46.

Sorof, S., Young, E. M., McCue, M. M. and Feterman, P. L.—(1963) *Cancer Res.*, **23**, 864.